# Synergy among Microbiota and Their Hosts: Leveraging the Hawaiian Archipelago and Local Collaborative Networks To Address Pressing Questions in Microbiome Research

**DOI:** 10.1128/mSystems.00159-17

**Published:** 2018-03-13

**Authors:** Nicole A. Hynson, Kiana L. Frank, Rosanna A. Alegado, Anthony S. Amend, Mohammad Arif, Gordon M. Bennett, Andrea J. Jani, Matthew C. I. Medeiros, Yuriy Mileyko, Craig E. Nelson, Nhu H. Nguyen, Olivia D. Nigro, Sladjana Prisic, Sangwoo Shin, Daisuke Takagi, Samuel T. Wilson, Joanne Y. Yew

**Affiliations:** a Department of Botany, University of Hawai‘i at Mānoa, Honolulu, Hawai‘i, USA; b Pacific Biosciences Research Center, University of Hawai‘i at Mānoa, Honolulu, Hawai‘i, USA; c Center for Microbial Oceanography: Research and Education, Department of Oceanography, University of Hawai‘i at Mānoa, Honolulu, Hawai‘i, USA; d Department of Plant and Environmental Protection Sciences, University of Hawai‘i at Mānoa, Honolulu, Hawai‘i, USA; e Department of Mathematics, University of Hawai‘i at Mānoa, Honolulu, Hawai‘i, USA; f Sea Grant College Program, University of Hawai‘i at Mānoa, Honolulu, Hawai‘i, USA; g Department of Tropical Plant and Soil Sciences, University of Hawai‘i at Mānoa, Honolulu, Hawai‘i, USA; h Department of Microbiology, University of Hawai‘i at Mānoa, Honolulu, Hawai‘i, USA; i Department of Mechanical Engineering, University of Hawai‘i at Mānoa, Honolulu, Hawai‘i, USA

**Keywords:** ecology, environmental microbiology, evolution, microbiome, paradigm shift

## Abstract

Despite increasing acknowledgment that microorganisms underpin the healthy functioning of basically all multicellular life, few cross-disciplinary teams address the diversity and function of microbiota across organisms and ecosystems. Our newly formed consortium of junior faculty spanning fields such as ecology and geoscience to mathematics and molecular biology from the University of Hawai‘i at Mānoa aims to fill this gap. We are united in our mutual interest in advancing a new paradigm for biology that incorporates our modern understanding of the importance of microorganisms. As our first concerted research effort, we will assess the diversity and function of microbes across an entire watershed on the island of Oahu, Hawai‘i. Due to its high ecological diversity across tractable areas of land and sea, Hawai‘i provides a model system for the study of complex microbial communities and the processes they mediate. Owing to our diverse expertise, we will leverage this study system to advance the field of biology.

## Abstract (precis)

Despite increasing acknowledgment that microorganisms underpin the healthy functioning of basically all multicellular life, few cross-disciplinary teams address the diversity and function of microbiota across organisms and ecosystems. Our newly formed consortium of junior faculty spanning fields such as ecology and geoscience to mathematics and molecular biology from the University of Hawai‘i at Mānoa aims to fill this gap.

## A SCIENTIFIC IMPERATIVE

Crucial to science is understanding whether it is itself reductionist: do first principles from one field, such as physics, explain the fundaments of others like molecular biology? The answer to this question hinges upon whether scientists speak the same language. Consortia that link multiple scientific fields offer the powerful promise of extending any single individual’s research to a more globally unifying endeavor. We see this as one of the potential primary outcomes of our newly established collaboration of junior faculty, the Center for Microbiome Analysis through Island Knowledge and Investigation (C-MAIKI). By overcoming the technical and philosophical barriers that often silo disparate disciplines, our goal is to advance the field of microbial systems biology. We are a diverse group of early career researchers from the University of Hawai‘i at Mānoa who are fascinated by the microbial world. Our individual research programs span natural environments, including sea, land, and freshwater, as well as model laboratory systems, such as fruit flies and rodents, while our study organisms range from viruses, bacteria, protists, and fungi to plants and animals, including humans (1–6). Some of us are not even biologists in the traditional sense, but rather mechanical engineers and mathematicians who look to nature to understand and create complex systems (7–9). Together, we seek to illuminate the diversity and function of microbes within Hawaiian ecosystems to examine whether their roles are conserved across hosts and environments and the extent to which disturbances alter the functioning of microbially mediated processes.

## PLAYING CATCH-UP: A CRITICAL NEED FOR BASIC MICROBIOME RESEARCH

While concepts such as keystone species, ecosystem engineers, and diversity/productivity relationships have been well documented for many macroorganisms, our knowledge of these features of microbial communities is limited. Three of the persistent challenges to assessing the ecology of microbes have been (i) ascribing meaningful taxonomic units that can be used to test evolutionary and ecological theories, (ii) disentangling the relative contributions of evolutionary and ecological processes to the community assembly and function of microbes, and (iii) understanding the spatial and temporal scales that are relevant for microbial interactions and the processes they mediate (10). Given the ever-increasing amounts of genomic data that can be used to determine the identity of microbes and the fact that evolutionary and population genetics theories provide fundamental frameworks for examining ecological patterns, solving the first two challenges seem relatively close at hand: it is just a matter of choosing a biological scale that is relevant for microbes (that is, what is the appropriate unit of selection for microorganisms?). Thus, we see the third challenge—deriving the appropriate space-time scales for the study of microbes, their interactions with each other, and their environments—as the most critical. We intend to make the most progress toward addressing this challenge in the years to come.

## HAWAI‘I AS A MODEL SYSTEM FOR THE STUDY OF MICROBIOMES

With our collective recognition that microbes underpin the healthy function of all multicellular life, we have come together to take a multifaceted approach to bridging two vast knowledge gaps in the quickly growing field of microbiome research: which environments act as reservoirs for symbiotic microbes when they are outside the host, and how we can manage our environments to promote and maintain healthy microbial partnerships? We are uniquely positioned to address these questions due to our diverse and complementary expertise, as well as our location on the island of O’ahu in the Hawaiian archipelago. Hawai‘i has long been recognized by climatologists, ecologists, and evolutionary biologists as a model system for the study of natural phenomena. While covering only 0.004% of the Earth’s total land area (similar to the size of Massachusetts), over a matter of kilometers, Hawaiian bioregions contain comparable environmental diversity to that typically found on much larger spatial scales, like continents. For example, Hawai‘i has a rainfall gradient from 204 mm/year on average near the summit of Mauna Kea (similar to averages in the Gobi Desert in Asia), to 10,271 mm/year on average on the slopes of Haleakalā (similar to parts of the Chocó-Darién, Columbia, one of the wettest places on Earth). Because Hawai‘i is one of the most remote island chains on Earth, non-human-assisted dispersal is a significant hurdle for the establishment of most organisms. As a result, Hawai‘i’s macrobiota is >90% endemic, with well-known evolutionary histories. While far less is known about the diversity of Hawai‘i’s microbes, evidence from recent surveys suggests that its microbial communities are dominated by taxa, at least to the generic level, found throughout the world (11, 12). For example, a recent study of phyllosphere fungi associated with a single genus of endemic Hawaiian plant *Clermontia* revealed that communities are hyperdiverse, harboring thousands of fungal operational taxonomic units (species equivalents). This result is similar to the findings of a global assessment of foliar fungal endophytes where tropical ecosystems harbored the greatest relative diversity (12, 13). Thus, Hawai‘i is a relatively small and closed system, with suites of environmental conditions similar to those found throughout the globe, and is occupied by many of the same guilds of microorganisms also found elsewhere. We have the ideal “living laboratory” for the study of microbiota and microbially mediated processes.

## FORGING AHEAD WITH THE STUDY OF HAWAIIAN MICROBIOTA

**FIG 1:**
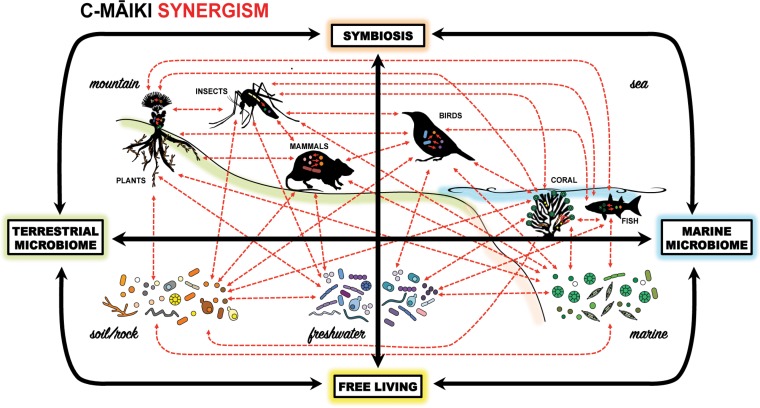
Conceptual schematic of C-MAIKI’s approach to address pressing questions in microbiome research by evaluating synergy (red arrows) among environmental microbiota and their hosts—from free-living to symbiotic lifestyles—leveraging the unique and replicable gradients across Hawaiian watersheds—from mountain (terrestrial) to sea (marine) habitats. Recognizing that microbes underpin the healthy function of all multicellular life, this synergistic cross-disciplinary approach allows us to address what environments act as reservoirs for symbiotic microbes when they are outside the host and how we can manage our environments to promote and maintain healthy microbial partnerships.

While prior research on microbiomes has focused on changes in microbial diversity across various environmental conditions in single hosts, we now recognize the connectivity across habitats and hosts and the microbial “neighborhood” as critical drivers of community composition (14) (Fig. 1). Through broad-scale sampling, we have identified traits and taxonomy that predict nonrandom overlap of microbial community members across disparate hosts and habitats (15, 16). For example, fungi in the genus *Malassezia* have been identified as numerically dominant in most marine systems examined throughout the Hawaiian Archipelago, yet some of the same genotypes appear in soil, plant, and even air samples (11). Also, studies on Hawaiian insect-microbe interactions have revealed that many host taxa selectively harbor distinct microbial communities, some of which appear to be intrinsic to hosts’ use of environmental resources and underpin their adaptation to Hawai‘i’s diverse environments (19). This connectivity begs the following questions: what habitat(s) represent the regional species pool, and are there conserved functions of these shared microbes across hosts and habitats?

As a next step to elucidate the mechanisms underlying these patterns of connectivity, we plan to assess the community dynamics of microbes as they transition from free-living to symbiotic states across an entire watershed, including the macrobiota living in it, that spans mountain ridges to fringing coral reef habitats (Fig. 1). Using a diversity of -omic approaches, as well as assays such as extracellular enzyme and lipid production to assess microbial functional traits, we will determine the identity and functional flexibility of microbes across hosts and habitats and how this impacts host and environmental health. Through manipulative field and laboratory experiments within and outside hosts and across steep environmental gradients, we will then test the resilience of microbial communities to perturbations in their living conditions. These data will provide the training ground for predictive probabilistic models that identify how we may manipulate microbial communities to restore or elicit specific host functions or ecosystem states. Using population genomic approaches combined with classic tools from population genetics and coalescent theory, we hope to advance our understanding of microbial migration and source-sink relationships for microbes, including how transitions from drastically different environments (e.g., terrestrial to marine) or hosts (e.g., corals to insects) are made.

## NO LONGER THE BLACK BOX: THE FUTURE IS BRIGHT FOR MICROBIOLOGY

Over the next 5 years, the field of microbiology has much to contribute, as well as much to learn, from the broader scientific community and disciplines. In the same vein as Thomas Kuhn’s *The Structure of Scientific Revolutions* (17), we would argue that the field of biology, until fairly recently, was in a stage of “normal science” where our interest in, and understanding of, the microbial world had been observed through the lens of macrobiology. Brought on by the immense technological innovations in DNA sequencing, we are shining a bright floodlight on the previously hidden world of microbes and its fundamental role in shaping ecosystems across all trophic levels. We are now in the early stages of a paradigm shift, the boundaries of which are only beginning to be delineated. In the years to come, we anticipate this paradigm shift being fueled by novel technologies and their ease of use, such as single-molecule sequencing, single-cell sorting from environmental communities, and increasingly streamlined computational platforms for large data sets. However, for this shift to become fully realized, it will not only require that we integrate microbes into (macro)biological concepts, but that we create new theories, models, and collaborations. As a cohort, the C-MAIKI faculty envisions the development of a new framework for biology that leverages our study system and our cross-disciplinary interactions to lay this new foundation.

## COLLABORATIONS, LIKE MICROBIOMES, ARE MORE THAN THE SUM OF THEIR INDIVIDUAL PARTS

On the flip side of reductionism is synergism, or the concept popularized by the field of ecology, that the additive effects of interacting organisms result in something more than the sum of their individual constituents. The classic example of synergism is the significant and positive relationship between plant diversity and primary productivity (18). While the cause for such synergism even among well-studied macroorganisms still remains somewhat elusive, our research endeavors will shed new light on the emergent properties of microbiomes. For example, collaborations among those of us who focus on model systems and those who work in field settings can transform our understanding of basic physiological and evolutionary processes. By using well-studied organisms such as *Drosophila* spp. in manipulative field experiments, we can elucidate the molecular mechanisms by which environmental microbes alter the physiology and phenotype of their hosts. Our collaborations will not only build a means for cross-communication among disciplines, perhaps revealing the reductionist side of microbiomes, but they will also result in synergistic breakthroughs that allow us to answer some of the most pertinent questions in the life sciences.
